# ADAM12 is a circulating marker for stromal activation in pancreatic cancer and predicts response to chemotherapy

**DOI:** 10.1038/s41389-018-0096-9

**Published:** 2018-11-16

**Authors:** V. L. Veenstra, H. Damhofer, C. Waasdorp, L. B. van Rijssen, M. J. van de Vijver, F. Dijk, H. W. Wilmink, M. G. Besselink, O. R. Busch, D. K. Chang, P. J. Bailey, A. V. Biankin, H. M. Kocher, J. P. Medema, J. S. Li, R. Jiang, D. W. Pierce, H. W. M. van Laarhoven, M. F. Bijlsma

**Affiliations:** 1 Amsterdam UMC, University of Amsterdam, LEXOR, Center for Experimental and Molecular Medicine, Cancer Center Amsterdam, Amsterdam, Netherlands; 2 Oncode Institute, Meibergdreef 9, 1105AZ Amsterdam, Netherlands; 3 Amsterdam UMC, University of Amsterdam, Department of Surgery, Cancer Center Amsterdam, Amsterdam, Netherlands; 4 Amsterdam UMC, University of Amsterdam, Department of Pathology, Cancer Center Amsterdam, Amsterdam, Netherlands; 5 Amsterdam UMC, University of Amsterdam, Department of Medical Oncology, Cancer Center Amsterdam, Amsterdam, Netherlands; 6 Wolfson Wohl Cancer Research Centre, Institute of Cancer Sciences, University of Glasgow, Glasgow, UK; 7 West of Scotland Pancreatic Unit, Glasgow Royal Infirmary, Glasgow, UK; 8 Centre for Tumour Biology, Barts Cancer Institute, Queen Mary University of London, London, UK; 9 Celgene Corporation, Summit, NJ USA; 10 Present Address: Biotech Research and Innovation Centre, University of Copenhagen, Copenhagen, Denmark

## Abstract

Pancreatic ductal adenocarcinoma (PDAC) is characterized by abundant stroma that harbors tumor-promoting properties. No good biomarkers exist to monitor the effect of stromal targeting therapies or to predict response. We set out to identify such non-invasive markers for PDAC stroma and predict response to therapy. Gene expression datasets, co-culture experiments, xenografts, and patient samples were analyzed. Serum samples were measured from a cohort of 58 resected patients, and 87 metastatic or locally advanced PDAC patients. Baseline and follow-up levels were assessed in 372 additional metastatic PDAC patients who received nab-paclitaxel with gemcitabine (*n* *=* 184) or gemcitabine monotherapy (*n* *=* 188) in the phase III MPACT trial. Increased levels of ADAM12 were found in PDAC patients compared to healthy controls (*p* < 0.0001, *n* *=* 157 and *n* *=* 38). High levels of ADAM12 significantly associated with poor outcome in resected PDAC (HR 2.07, *p* *=* 0.04). In the MPACT trial survival was significantly longer for patients who received nab-paclitaxel and had undetectable ADAM12 levels before treatment (OS 12.3 m vs 7.9 m *p* = 0.0046). Consistently undetectable or decreased ADAM12 levels during treatment significantly associated with longer survival as well (OS 14.4 m and 11.2 m, respectively vs 8.3, *p* *=* 0.0054). We conclude that ADAM12 is a blood-borne proxy for stromal activation, the levels of which have prognostic significance and correlate with treatment benefit.

## Introduction

Survival of patients diagnosed with pancreatic ductal adenocarcinoma (PDAC) has remained poor for many decades^1^. Median survival after diagnosis is around 6 months, and even patients treated with a combination of surgery and chemotherapy typically succumb to the disease within 5 years^2^. The vast majority of patients are diagnosed with advanced disease, precluding resection of the tumor. Treatment options for these patients are limited and clinical trials typically demonstrate only partial benefit, in a limited number of patients. Biomarkers that allow patient stratification and early response monitoring are expected to improve outcome.

In recent years, research on PDAC has shifted focus from tumor cells to the abundant non-malignant compartment known as the *stroma*. This stroma consists of extracellular matrix proteins, cancer- associated fibroblasts (CAFs), and immune cells^3^. The stroma typically forms the vast majority of tumor bulk and contributes to chemoresistance by acting as a barrier to the delivery of chemotherapeutics. Non-mechanical tumor promoting properties have been attributed to the stroma as well^4^.

Nab-paclitaxel is an albumin-bound nanoparticle formulation of paclitaxel that enhances distribution and penetration into tumor tissues^5,6^. While the initially suspected role for SPARC (which is expressed on stromal fibroblasts and binds albumin) in conferring enhanced sensitivity to nab-paclitaxel in PDAC was not confirmed^7,8^. The combination of nab-paclitaxel with gemcitabine is effective compared to gemcitabine monotherapy and median survival increases from 6.6 to 8.7 months^9^. Other stroma targeting agents have also been tested in trials, but these have failed to demonstrate favorable outcomes^10^. Given the practical hurdles to obtaining tissue from the pancreas for histopathological assessment, good non-invasive markers are urgently needed that inform on the PDAC stroma to allow selection of patients for stroma-targeting therapies, and/or to monitor the response to such regimens. Currently, there is a paucity of such markers^11^.

ADAM12 is a member of the A Disintegrin And Metalloproteases (ADAM) protein family and harbors extracellular metalloprotease, and intracellular signaling properties. The protein is involved in cell adhesion by binding to integrins and syndecans, as well as the proteolytic cleavage of substrates from producing cells, a process known as ectodomain shedding^12^. Cancer-related substrates of ADAM12 include epidermal growth factor (EGF), and Sonic Hedgehog (SHH)^13–15^. Expression of ADAM12 is elevated in glioblastoma, breast, bladder, lung, prostate, and liver cancer^16–21^. ADAM12 expression correlates with tumor stage in breast and bladder cancer, and is prognostic in small cell lung cancer^16,21,22^. A previous study showed the upregulation of ADAM12 in pancreatic CAFs compared to fibroblasts from non-neoplastic tissue, but the clinical or functional relevance was not explored^23^. Here, we show that ADAM12 is associated with the activated stroma, and poor-prognosis mesenchymal molecular subclasses, in PDAC. It is elevated in the serum of PDAC patients and associates with poor outcome. In an exploratory analysis of patients treated with nab-paclitaxel and gemcitabine, circulating ADAM12 levels predicted outcome.

## Results

### ADAM12 associates with activated pancreatic cancer stroma and poor-prognosis molecular subclasses

**Fig. 1:**
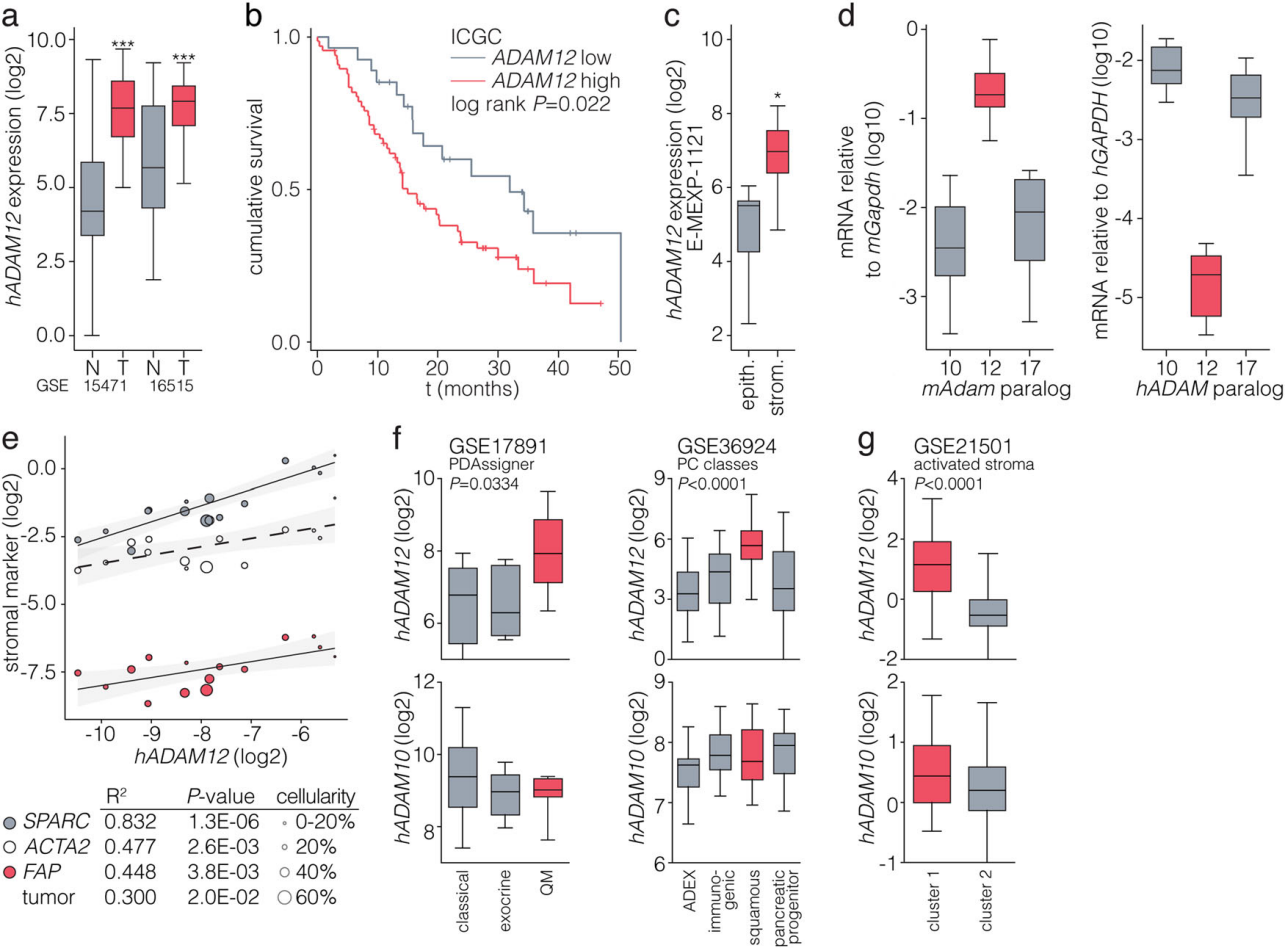
ADAM12 associates with activated pancreatic cancer stroma and poor-prognosis molecular subclasses. **a** Boxes indicate median with first and third quartiles of log2 transformed gene expression values from two U133 Plus 2.0 microarray datasets of pancreatic cancer patients comparing normal and tumor tissue. Badea et al. set (GSE15471), *n* *=* 36 paired biopsies; Pei et al. set (GSE16515); *n* *=* 16 (normal), *n* *=* 36 (tumor). ***p < 0.001, statistical testing was by two-tailed Student’s t test. **b** Kaplan–Meier analysis of patients from the ICGC cohort, dichotomized for median *ADAM12* expression^25^. **c** Log2 transformed *ADAM12* expression values from the Pilarksy et al. (E-MEXP-1121)^27^ gene expression set obtained from microdissected pancreatic cancer tissue. *p < 0.05, testing by two-tailed Student’s t test. **d** Transcript levels for indicated *Adam/ADAM* paralogs relative to *Gapdh/GAPDH* were measured in xenografts by qPCR using mouse- or human-specific primers. Boxplots show data from 10 individual patient grafts. For each replicate sample measured by qPCR, a technical triplicate was used. Difference between groups was tested by ANOVA for both panels *p* < 0.0001. **e** Association of *ADAM12* expression with stromal activation markers in the AMC patient cohort was measured by qRT-PCR, *n* *=* 15 patients. Size of dots indicates tumor cellularity scored by a pathologist. **f** Patients from GSE17891 dataset were grouped by published PDAssigner subtype labels and log2 expression of *ADAM12* and paralog *ADAM10* is shown (*n* *=* 27). Patients from GSE17891 were grouped by published PC class labels (*n* = 96). Difference between groups was tested by ANOVA. **g** Patients were k-means clustered using the activated stroma gene set and *ADAM12* and -*10* expression is shown (*n* *=* 132). Difference between groups was tested by ANOVA

We identified ADAM12 in a previous screen for stromal targets of tumor-derived SHH^24^. To confirm that ADAM12 is expressed in human pancreatic cancers, we queried publically available gene expression datasets that contain normal pancreas and pancreatic cancer tissue. *ADAM12* was significantly higher expressed in tumor tissue (Fig. 1a and Supplementary Fig. S1a), and high expression of ADAM12 was associated with worse survival following resection (Fig. 1b and Supplementary Fig. S1b)^25,26^. Microdissected tumor tissue expression data confirmed a predominantly stromal expression of *ADAM12* (Fig. 1c)^27^. To further delineate the source of *ADAM12* expression, we measured its expression by species-specific qPCR in patient-derived xenografts (PDXs)^28^. Mouse *Adam12* expression in stromal host cells was found to be high compared to other well-characterized paralogs (*Adam10* and -*17*; Fig. 1d)^14^.

To determine if ADAM12 expression is a hallmark of tumors with highly activated stromal stellate cells and (myo)fibroblasts, its correlation with known markers for such cells was determined by qPCR in bulk tumor tissue (Fig. 1e, and Supplementary Fig. S2a). A strong correlation of *ADAM12* was found with secreted protein acidic and cysteine rich (*SPARC*), α-smooth muscle actin (*ACTA2*), and fibroblast activation protein (*FAP*). No strong inverse correlations with tumor cellularity were found. Gene set enrichment analysis revealed a significant enrichment of extracellular matrix and stromal pathway signatures in patients with high *ADAM12* expression (Supplementary Fig. S2b).

Subclasses of PDAC have been defined at the gene expression level. All current classifications identify a subtype that is characterized by mesenchymal features and increased stromal infiltration^29^. We found that *ADAM12* expression associated with both the Collisson et al. quasi-mesenchymal^30^ and the Bailey et al. squamous subtype tumors^31^ (Fig. 1f). Patients clustered with the activated stroma signature from Moffit et al.^32^ also showed high expression of *ADAM12* (Fig. 1g). These analyses show that the expression of *ADAM12* associates with poor-prognosis mesenchymal subgroups of PDAC.

### ADAM12 expression is driven by tumor cell-derived TGF-β

**Fig. 2:**
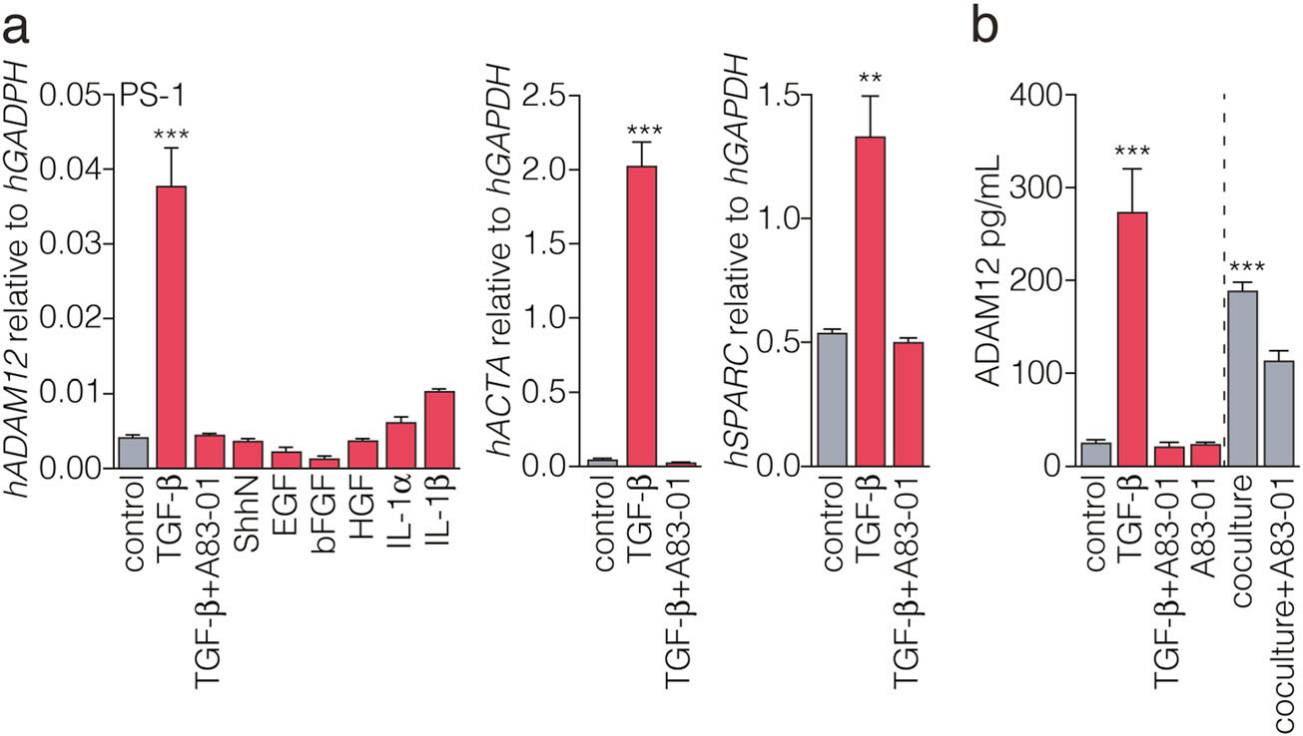
ADAM12 expression is induced by tumor cell-derived TGF-β. **a** Immortalized stellate cells (PS-1)^46^ were starved with 0.5% FCS for 24 h and subsequently treated with the indicated ligands for 48 h. Concentrations used: TGF-β, 5 ng/mL; TGF-β receptor I inhibitor A83-01, 1 µM; ShhN supernatant from 293T cells, 1:4; EGF, 50 ng/ml; bFGF, 10 ng/ml; HGF, 10 ng/ml; IL-1α, 10 ng/ml; IL-1β, 10 ng/ml. In addition to ADAM12, expression analysis of stromal activation marker genes ACTA and SPARC in response to TGF-β is shown. Two-tailed Student’s t test comparing control vs. TGF-β, ***p < 0.001, **p < 0.001. *n* = 6, across two separate experiments for control and TGF-b, *n* = 3 in one experiment for other treatments. For each replicate sample measured by qPCR, a technical triplicate was used. Bars show mean ± SEM. **b** To ascertain that tumor cell-derived TGF-β drives ADAM12 expression, stellate cells were treated as for panel **a**, or cocultured with tumor cells of the 84 primary culture. Supernatant was harvested and cleared by centrifugation. Soluble ADAM12 levels were measured by ELISA. At least three replicates are shown, statistical testing was by two-tailed Student’s t test comparing control vs. TGF-β, and control vs. coculture, ***p < 0.001. *n* = 6, across two separate experiments for treatments, n = 3 in one experiment for coculture. For each replicate sample measured by qPCR, a technical triplicate was used. Bars show mean ± SEM

Several tumor-derived signals have been identified that shape the stroma by activating the cells that reside in it. For instance, transforming growth factor beta (TGF-β) is a strong activator of CAFs and pancreatic stellate cells (PSCs) during cancer progression^33,34^. To functionally confirm this activation mechanism to drive ADAM12 expression, we treated human stellate cells with TGF-β and other ligands known to be involved in tumor-stroma crosstalk. An upregulation of ADAM12 was only apparent in stellate cells treated with TGF-β (Fig. 2a).

ADAM12 exists as soluble proteins^12^. These forms can be generated by shedding of the cell-bound protein, but in humans a soluble isoform (ADAM12-S) also exists. To determine if soluble ADAM12 is produced by activated stellate cells, PS-1 cells were stimulated using TGF-β or by coculturing with primary PDAC tumor cells, and ADAM12 was measured by ELISA in the supernatant of these cultures (Fig. 2b). As for the transcript analysis, a strong upregulation of ADAM12 was observed following TGF-β dependent activation of stellate cells. Coculture of human stellate cells with primary tumor cells led to a significant upregulation of soluble ADAM12 that could be blocked by the TGF-β pathway inhibitor A83-01, confirming that active TGF-β ligand is present in these cocultures and able to drive ADAM12 secretion in stromal cells^35^.

### ADAM12 is elevated in the serum of PDAC patients and predicts outcome after resection

**Fig. 3:**
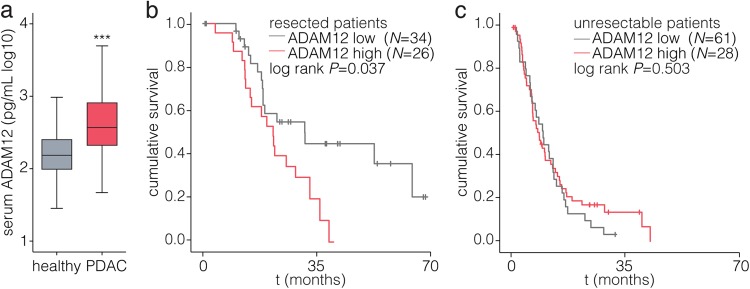
ADAM12 is elevated in the serum of PDAC patients and predicts poor outcome in patients undergoing resection. **a** ADAM12 levels were measured by ELISA in serum of healthy individuals (*n* *=* 38), and patients diagnosed with PDAC (*n* *=* 157). Boxplots show median with interquartile range. ***p < 0.0001; tested by Mann–Whitney U-test against healthy controls. **b** Kaplan-Meier survival analysis of AMC PDAC patients who underwent resection of the tumor, dichotomized by baseline serum levels determined by receiver-operator-characteristics (ROC; 316 pg/mL). **c** As for panel **b**, for patients who did not undergo resection. Statistical testing was by log-rank test

Having established the association of ADAM12 with stromal activation and poor-prognosis molecular subclasses, we proceeded to evaluate ADAM12 as a non-invasive biomarker in PDAC. Patients diagnosed with PDAC before therapeutic intervention showed a significant elevation of serum ADAM12 compared to healthy individuals (Fig. 3a). The association of serum ADAM12 levels with clinical parameters was analyzed (Supplementary Table S1). Patients were dichotomized using serum ADAM12 levels determined by receiver-operator-characteristics (ROC, for live-dead resected patients at time of analysis; 316 pg/ml). No correlations of serum ADAM12 with age, primary tumor size, and disease stage were found (Supplementary Table S2). High ADAM12 levels in the resected cohort associated with poor survival (HR = 2.07, *p* = 0.041), as did high CA19-9 levels and high LNR (Table 1). In a multivariate analysis no significant associations were found (Supplementary Table S3). The impact of serum ADAM12 on overall survival was analyzed by Kaplan–Meier analysis and log-rank test. We found that whereas serum ADAM12 did not significantly associate with survival in unresectable patients, in resected patients higher ADAM12 levels were strongly associated with shorter survival (Fig. 3b, c). It thus appears that activated stroma, as revealed by high serum ADAM12 levels, contributes to poor disease outcome when the tumor is at a resectable stage.

**Table 1:** Cox proportional hazard regression model

Univariate		Resected			Unresectable		
		HR	95% CI	*p*-value	HR	95% CI	*p*-value
Age		1.01	0.97–1.04	0.73	1.02	0.99–1.05	0.203
Gender	Male	1			1		
	Female	0.64	0.31–1.32	0.225	1.32	0.84–2.07	0.236
Stage	I	1					
	II	2.37	0.56–10.01	0.242	1		0.022
	III	2.14	0.41–11.12	0.368	1.44	0.59–3.54	0.424
	IV	3.86	0.35–42.93	0.273	2.51	1.06–5.99	0.038
Tumor size	≤ 20 mm	1			1		
	> 20 mm	2.01	0.83–4.92	0.124	25.36	0.37–1749	0.134
CA19–9	Low	1			1		
	High	3.28	1.05–10.19	0.04	2.11	1.21–3.69	0.008
LNR		7.49	1.61–34.89	0.01	n/a	n/a	n/a
ADAM12	Low	1			1		
	High	2.07	1.03–4.16	0.041	0.67	0.41–1.10	0.116

Univariate analysis for overall survival in PDAC patients (resected *N* = 58; unresectable *N* = 86). Dichotomization of resected patients by CA19-9 was at 415 kU/L, by ADAM12 at 316 pg/mL. Dichotomization of unresectable patients by CA19-9 was at 354 kU/L. LNR lymph node ratio

### ADAM12 levels predict favorable outcome in patients treated with nab-paclitaxel

The phase III MPACT trial showed survival benefit of nab-paclitaxel with gemcitabine compared to gemcitabine in metastasized PDAC patients, and is relatively well tolerated. To determine if ADAM12 levels associate with response to nab-paclitaxel, we measured its levels in plasma samples from the MPACT cohort^9,36,37^.

Baseline samples were measured and it was observed that the decreased sensitivity of detection in plasma (rather than serum) resulted in a considerable number of samples that had undetectable levels of ADAM12 as defined by 2 × standard deviation of the optical density of blanks. Dichotomization of the MPACT cohort by this cutoff resulted in groups with similar size across treatment arms and baseline characteristics (Supplementary Table S4 and S5) but significantly worse survival for patients with detectable ADAM12 (Supplementary Fig. S3a). Univariate Cox regression revealed a HR of 1.41 (1.10–1.81 95% CI; *p* = 0.0062) for detectable ADAM12 (Supplementary Table S6) in this cohort.

**Fig. 4:**
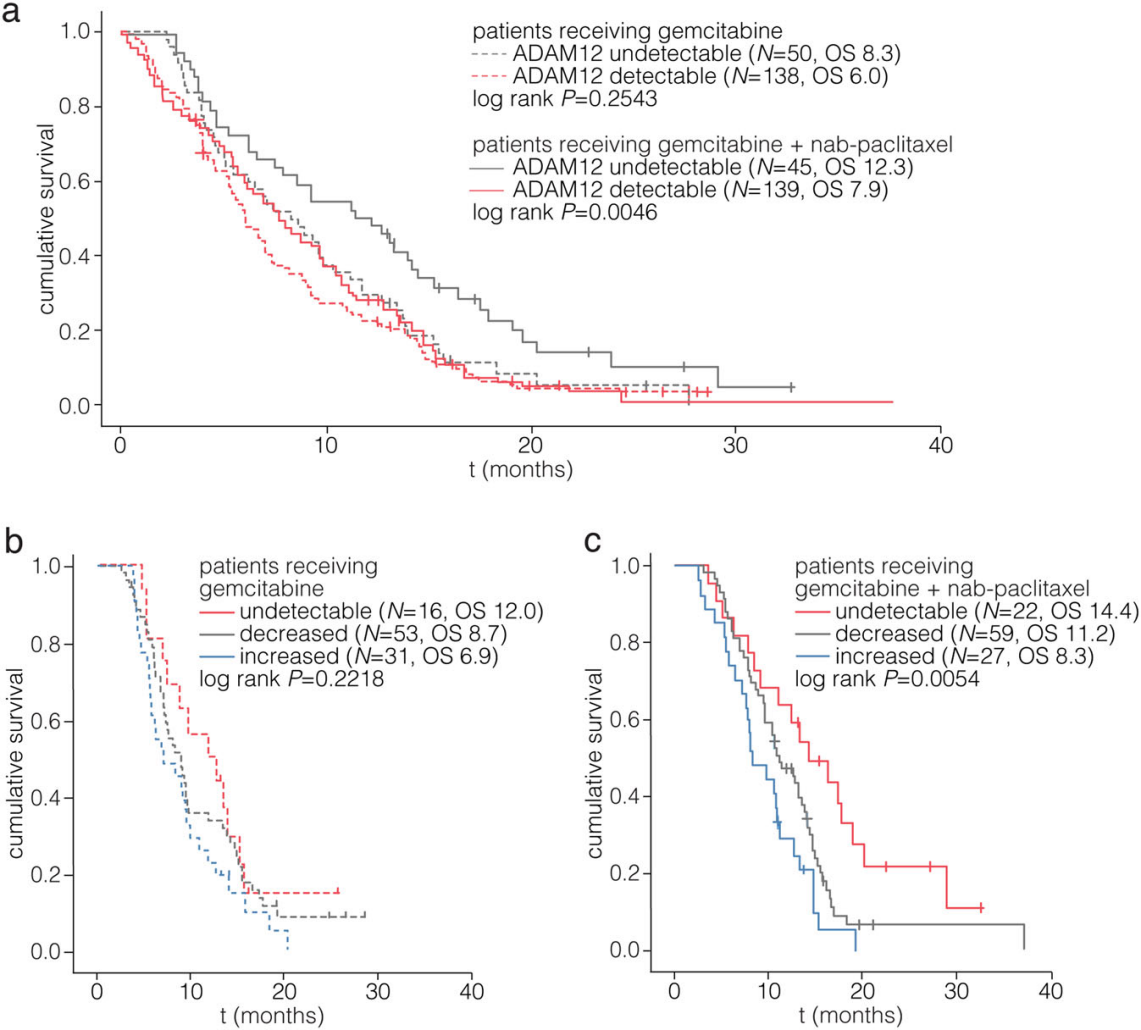
Plasma ADAM12 predicts favorable outcome in patients treated with nab-paclitaxel and gemcitabine. **a** Baseline recalcified plasma samples from the MPACT cohort were analyzed for ADAM12 and patients were dichotomized for ADAM12 levels above or below detection (2× SD of blanks). Patients in the arm receiving gemcitabine monotherapy are indicated by dashed lines. Numbers in parentheses indicate number of patients in the group (*n*), and median survival for that group (OS). Analysis of patients in the arm receiving gemcitabine and nab-paclitaxel is shown with solid lines. **b** Baseline (cycle 1, day 1) and follow-up (cycle 2, day 1) samples were measured and fold change was calculated. Samples with undetectable ADAM12 levels at both baseline and follow-up did not show a fold change and are indicated by the red line. Patients in the arm receiving gemcitabine monotherapy are shown. **c** As for panel **b**, analysis of patients in the arm receiving gemcitabine and nab-paclitaxel. Statistical testing in all panels was by log-rank test

When the trial arms were analyzed separately, ADAM12 levels did not significantly associate with survival in patients that received gemcitabine monotherapy (Fig. 4a, dashed lines, *p* = 0.2543). Conversely, in patients that received nab-paclitaxel with gemcitabine, undetectable plasma ADAM12 strongly associated with favorable outcome (solid lines, *p* = 0.0046). Patients with undetectable ADAM12 showed a median survival benefit of over 4.0 months from the addition of nab-paclitaxel to gemcitabine, as compared to a benefit of 1.9 months for patients with detectable ADAM12. Baseline ADAM12 levels were significantly associated with outcome in a multivariate model including KPS, and treatment as factors (Supplementary Table S7). Inclusion of CA19.9 in the model yielded a non-significant association of ADAM12 with survival.

Next, we determined the predictive power of the change in ADAM12 levels during treatment from cycle 1, day 1 (baseline) to cycle 2, day 1 (follow-up) samples (Supplementary Table S4b, baseline characteristics in Supplementary Table S5b). A reduction in ADAM12 levels is likely to be caused by a diminished stromal activation, or a reduced tumor load. Indeed, a reduction in plasma ADAM12 associated with improved survival (Supplementary Fig. S3b). Importantly, these associations were all driven by the favorable outcome of patients with reduced (OS 11.2 m) or repeatedly undetectable (OS 14.4 m) ADAM12 in the nab-paclitaxel with gemcitabine-treated trial arm, as compared to the increased levels (OS 8.3) of ADAM12 (Fig. 4b, c). Although statistical significance was not reached in the gemcitabine arm, the rank ordering of the groups defined by change in ADAM12 levels on treatment was the same in both arms of the trial. In the poorest-outcome group (in which ADAM12 became detectable or increased at cycle 2), the overall survival was numerically superior at 8.3 months in the nab-paclitaxel with gemcitabine arm as compared to the monotherapy arm at 6.9 months. Thus, our results do not indicate an absence of treatment benefit from nab-paclitaxel in the patients with high or increased ADAM12.

In conclusion, we established that ADAM12 is a serum-borne proxy for the stromal activation of pancreatic cancers, and that its levels associate with poor disease outcome. A low level of or decrease in ADAM12 is associated with improved survival in patients treated with nab-paclitaxel and gemcitabine, and could possibly be used to stratify patients in future trials using this or other treatment combinations.

## Discussion

PDAC is characterized by an abundance of activated stroma that harbors both tumor promoting, as well as suppressive properties^38–40^. Here, analysis of gene expression data, patient-derived xenografts and co-culture experiments identified ADAM12 as a circulating marker for activated stroma and tumors of poor-prognosis molecular subclasses. In patients with resectable disease, high ADAM12 levels predicted inferior survival outcomes. This could imply that ADAM12 is not a general stromal activation marker, but that there are very specific biological programs affected by this protein. Known roles for stromal ADAM12 are manifold: the well characterized function of ADAM12 as a sheddase of growth factor receptor ligands such as EGF can provide a nurturing environment for tumor cells^41,42^. Furthermore, ADAM12 has been shown to participate in matrix remodeling by cleavage of extracellular matrix proteins, suggesting involvement in cancer invasion and metastasis formation^21^. Further studies, similar to ones performed for breast and prostate cancer, are needed to elucidate the functional contributions of ADAM12 to pancreatic cancer.

In metastatic PDAC patients treated with gemcitabine, ADAM12 was not associated with survival. However, low ADAM12 levels did predict response to treatment in the cohort of patients treated with nab-paclitaxel and gemcitabine. This suggests that the supportive role of the stroma in the metastases predominates, to induce resistance to cytotoxic treatment. In this case, low ADAM12 levels identify lesions that are more sensitive to cytotoxic treatment. Clearly, such an effect becomes more apparent in effective treatment regimens, such as nab-paclitaxel with gemcitabine. Future studies are needed to show that ADAM12 retains its predictive power for other effective cytotoxic treatment regimens, such as FOLFIRINOX^43^.

Alternatively, it could be argued that the stroma-targeting properties of nab-paclitaxel explain why low ADAM12 predicts favorable outcome of nab-paclitaxel and gemcitabine. Nab-paclitaxel is accepted to have at least some degree of specificity for the stroma: the conjugation to albumin has been suspected to increase taxane concentration at sites of high SPARC (an albumin receptor) expression such as stromal (myo)fibroblasts^6^. In tumors with relatively few activated stromal cells, nab-paclitaxel will therefore be able to effectively eradicate the vast majority of the stroma, whereas in tumors with highly abundant stroma, the ablation is only partial and not effective in the long term.

One limitation of the current study is the exploratory, retrospective nature of this analysis. The MPACT trial was not statistically powered to test the interaction between serum markers such as ADAM12 and treatment groups. Furthermore, it is possible that the predictive value of ADAM12 as inferred from the differences in response to treatment is a result of the longer survival of patients receiving nab-paclitaxel with gemcitabine. Moreover, it should be noted that other biomarker discovery studies have not yet yielded applicable markers. For instance the initially suspected role for SPARC in conferring enhanced sensitivity to nab-paclitaxel in PDAC was not confirmed^7,8^, and its expression did not correlate with response to nab-paclitaxel with gemcitabine. Decreased CA19-9 levels were initially described to associate with response rate in the experimental arm of the MPACT trial^37^, and high baseline CA19.9 was associated with reduction in risk of death from the combination treatment (gemcitabine with nab-paclitaxel)^9^. However, in a follow-up analysis CA19-9 levels did not predict survival in the combination treatment arm^36^. Other studies have identified panels of serum-borne markers for PDAC stroma and correlated these to response in experimental models for, but so far, predictive value has not been assessed^11^.

Although the significant association of serum ADAM12 with outcome was not sustained in multivariate analysis of the MPACT samples when CA19-9 was included in our model, levels of ADAM12 were very strongly associated with favorable responses to nab-paclitaxel with gemcitabine suggesting that the analysis of a sufficiently large cohort could confirm predictive power. This will offer important possibilities for patient stratification. ADAM12, or similar blood-borne proxies for stromal activation could possibly be used to stratify patients in future trials with experimental stroma targeting agents, as well as for currently used (chemo)therapeutics. In summary, we have shown that ADAM12 expression in pancreatic cancer is indicative of highly activated stroma as well as poor-prognosis mesenchymal subclasses of tumors. Systemic levels of ADAM12 were found to be strongly prognostic in resectable PDAC patients, and low ADAM12 levels were associated with improved survival in patients treated with nab-paclitaxel and gemcitabine. ADAM12, or similar blood-borne proxies for stromal activation could possibly be used to stratify patients in future trials with experimental stroma targeting agents, as well as for currently used (chemo)therapeutics. To definitively establish the predictive power of ADAM12 or similar proxies for stromal activation in solid cancers, we propose a prospective clinical trial, using the levels of such markeres as intervention to stratify patients for currently available (chemo)therapeutics, but also experimental stroma targeting agents.

## Materials and methods

### Collection of blood samples

In the AMC cohort, serum samples were obtained perioperatively from 60 patients undergoing resection, or before the start of treatment in case of unresectable patients (*n* = 89). Clinicopathological data were obtained from medical records and included age, gender, tumor diameter, differentiation grade, lymph node ratio (positive lymph nodes/total number of lymph nodes examined), therapies received, and tumor-node-metastasis (TNM) staging. Collection of material was approved by the institute’s ethics committee (IRB), and written informed consent was received from all participants (AMC METC 2014_181)^28^. Blood samples of 38 non-age matched healthy individuals without any indication of malignancy were collected as a control group. In the MPACT trial cohort, collection of plasma samples for biomarker development was optional and separate written consent was obtained for sample collection and biomarker analysis. The patients and methods of the MPACT trial have been described previously (trial registration number NCT00844649)^37^. All patients provided infromed consent. In brief, patients were ≥ 18 years of age, had confirmed measurable metastatic adenocarcinoma of the pancreas, a Karnofsky performance status (KPS) of ≥ 70, and did not receive prior chemotherapy for metastatic disease. Patients were randomized 1:1 (stratified by performance status, presence of liver metastases, and region) to receive *nab*-paclitaxel 125 mg/m^2^ plus gemcitabine 1000 mg/m^2^ on days 1, 8, and 15 of every 28 days or gemcitabine alone 1000 mg/m^2^ on days 1, 8, 15, 22, 29, 36, and 43 in 56 days (cycle 1) and then on days 1, 8, and 15 of every 28 days (cycle ≥ 2). Treatment continued until disease progression or unacceptable toxicity.

### Establishment of patient-derived xenografts and primary cell lines

Collection of tumor material was approved by the institute’s ethics committee in accordance with the Declaration of Helsinki (AMC METC 2014_181). Written informed consent was received from all participants^28^. All PDAC patients treated in the Academic Medical Center were diagnosed by pathology or cytology. Specimens were processed and inspected according to national and international guidelines. An experienced pathologist performed microscopic assessment. Final diagnosis was set in accordance with the WHO classification, and the pTNM classification of malignant tumors. Freshly excised tumor pieces (approximately 3 × 3 × 3 mm) originating from the primary tumor or liver metastasis were washed several times in PBS containing 10 µg/ml gentamycin (Lonza, Basel, Switzerland) and 1% penicillin-streptamycin. Pieces were grafted subcutaneously into the flank of immunocompromised NOD.Cg-Prkdc^scid^ Il2rg^tm1Wjl^/SzJ (NSG) mice (JAX 005557) with Matrigel (BD, Franklin Lakes, NJ). Both sexes were used, and ages ranged up to 9 months. Animals were bred and maintained at the local animal facility according to the pertinent legislation, and ethical approval was obtained for all procedures (DTB/LEX102348). After outgrowth to a size of ∼500 mm^3^, tumors were harvested and passaged, and/or used to establish in vitro cultures. For this, harvested xenografts were minced with a scalpel blade, placed in IMDM with 8% FBS and collagenase IV (0.5 mg/ml, Sigma-Aldrich, St. Louis, MO) and incubated at 37 °C for 60 min with vortexing every 15 min. The dissociated suspension was passed through a 70 µm cell strainer, washed with culture medium and grown in IMDM containing 8% FBS and 50 µM β-mercaptoethanol. During the first 5–10 passages, cultures contained colonies of human epithelial cells and a layer of murine fibroblasts. A culture without epithelial component, as confirmed by flow cytometry using an anti-EPCAM antibody (DAKO, F0860 at 1:100), was used for stimulation experiments. STR profiling was performed to confirm donor-cell line matching (August 2017). For transcript analysis, all eligible and available xenografts were analyzed. Randomization and blinding did not apply. Xenografts were excluded if histopathological assessment did not confirm diagnosis of PDAC.

### RNA isolation and quantitative real-time PCR

Small pieces of PDX tumor were homogenized using an Ultra-Turrax tissue homogenizer T8 (IKA-Werke, Staufen im Breisgau, Germany) in 1 ml of Trizol (ThermoFisher). Primary cells were lysed in Trizol and RNA isolation was performed according to the manufacturer’s protocol. Snap frozen patient tumor samples were embedded in Tissue-Tek OCT (Sakura FineTek, Japan) and 30 slices of 20 µm each were cut on a cryotome. Cut tissue was immersed in 1 ml of RNA Bee (Amsbio, Abingdon, United Kingdom), homogenized, and RNA isolation was performed according to manufacturer’s protocol (Qiagen, Hilden, Germany). For tumor percentage scoring, a 10 µm slice was kept before the tissue was cut in 20 µm slices, and processed for H&E staining. Scoring of tumor percentage was performed by an experienced pathologist. cDNA was synthesized using Superscript III (ThermoFisher) and random primers. Real-time quantitative RT-PCR was performed with SYBR green (Roche, Basel, Switzerland) on a Lightcycler LC480 II (Roche). Relative expression of genes was calculated using the comparative threshold cycle (Cp) method and values were normalized to reference gene GAPDH/Gapdh. Primer sequences are: *hGAPDH* Fw 5′ gaaggtgaaggtcggagtc 3′; *hGAPDH* Rv 5′ tggaagatggtgatgggatt 3′; *hADAM10* Fw 5′ ttcgatgcaaatcaaccaga 3′; *hADAM10* Rv 5′ ttccttcccttgcacagtct 3′; *hADAM12* Fw 5′ tttccaccaccctctcagac 3′; *hADAM12* Rv 5′ gcctctgaaactctcggttg 3′; *hADAM17* Fw 5′ gggaacatgaggcagtctct 3′; *hADAM17* Rv 5′ accgaatgctgctggatatt 3′; *hACTA2* Fw 5′ caaagccggccttacagag 3′; *hACTA2* Rv 5′ agcccagccaagcactg 3′; *hFAP* Fw 5′ tcagtgtgagtgctctcattgtat 3′; *hFAP* Rv 5′ gctgtgcttgccttattggt 3’; *hSPARC* Fw 5’ gaaagaagatccaggccctc 3′; *hSPARC* Rv 5′ cttcagactgcccggaga 3′; *hKRT19* Fw 5′ cctggagttctcaatggtgg 3′; hKRT19 Rv 5′ ctagaggtgaagatccgcga 3′; mGapdh Fw 5′ ctcatgaccacagtccatgc 3′; *mGapdh* Rv 5′ cacattgggggtaggaacac 3′; *mAdam10* Fw 5′ aagatggtgttgccgacagt 3′; *mAdam10* Rv 5′ tggtcctcatgtgagactgc 3′; *mAdam12* Fw 5′ gctttggaggaagcacagac 3′; *mAdam12* Rv 5′ cgcatcaacgtcttcctttt 3′; *mAdam17* Fw 5′ gtacagcgtgaagtggcaga 3′; *mAdam17* Rv 5′ gccccatctgtgttgattct 3′.

### Gene set enrichment analysis (GSEA) and expression analysis

Gene set enrichment analysis (v2.0.14) software was downloaded from the Broad Institute website (http://www.broadinstitute.org/gsea) and used according to the author’s guidelines^44^. Median ADAM12 expression was used to dichotomize samples. Gene set for the GO term ‘extracellular matrix’ was downloaded from the Molecular Signature Database (MSigDB; V4.0); the pancreatic stroma signature was published by Binkley et al.^45^ 2000 phenotype permutations were used to determine significance of the enrichment score. Gene expression data were collected and processed for use in the AMC in-house R2 Genomics Analysis and Visualization Platform: (http://r2.amc.nl). For visualization of gene expression, data were imported in R and plotted using ggplot2, or plotted in Graphpad Prism.

### Treatment of cultures

Stellate cells (from the Kocher lab^46^) were seeded in 12-well culture plates and upon reaching confluence, pre-starved overnight in 0.5% FBS containing medium and treated for 24 h with 5 ng/ml recombinant human TGF-β1 (Peprotech, London, UK) in the presence or absence of 1 µM ALK4/5/7 inhibitor A83-01 (Tocris Bioscience, Bristol, UK). ShhN supernatant from 293T cells was added 1:4, EGF was used at 50 ng/ml, bFGF, 10 ng/ml; HGF 10 ng/ml; IL-1α, 10 ng/ml; IL-1β, 10 ng/ml. Treatment was not randomized between plate wells, and experiments were not blinded. Cultures were STR authenticated (June 2016), and tested for mycoplasma monthly.

### ELISA analysis of serum samples (AMC cohort)

For the reporting on association of ADAM12 levels with prognosis, the pertinent guidelines were considered^47^. Serum was obtained by centrifugation of blood for 10 min at room temperature at 1300 × *g*, and storage at −80 °C until analysis. ADAM12 levels were determined using the human ADAM12 DuoSet ELISA kit (R&D Systems, Minneapolis, MN), according to manufacturer’s recommendations. Briefly, after coating the 96-well plates (Greiner Nunc MaxiSorp, Kremsmünster, Austria) with capture antibody overnight at room temperature, blocking the plate with 1% BSA the following day, 80 µl of serum samples were added. After incubation for two hours at room temperature and mild washing steps, samples were incubated with biotinylated detection antibody for additional two hours followed by a 20 min incubation step with horse-radish peroxidase (HRP)-labeled streptavidin. Substrate was tetramethylbenzidine substrate solution (TMB), added for an additional 20 min. Absorbance was measured at 450 nm and 570 nm with a microplate reader (BioTek Synergy BioTek, Winooski, VT) after addition of 1 M H_2_SO_4_ stop solution. For wavelength correction, 570 nm readings were subtracted from the 450 nm values before further analysis. The technician performing the analysis was blinded for survival time. Randomization did not apply.

### ELISA analysis of plasma samples (MPACT cohort)

MPACT trial blood samples were collected in EDTA tubes to chelate calcium and prevent blood clotting, and storage at −80 °C until analysis. To allow the analysis of these plasma samples using the ADAM12 DuoSet ELISA, recalcification was required. Hundred microliters of EDTA plasma was complemented with 12 mM CaCl_2_ (final concentration) and incubated overnight at 4 °C. The following day, clots were removed manually and 50 µl sample was used for the assay. The ELISA was performed as for the serum samples. Analysis was blinded to the technician performing the ELISA, who was unaware of the trial arm the samples were from. Sensitivity of the assay was reduced compared to measurement in serum by a factor of 0.44, explaining why in the MPACT cohort, the lowest quartile was composed of samples that had undetectable plasma ADAM12. Measurement of ADAM12 in matched serum and plasma samples revealed high correlation (R^2^ = 0.9971, *p*-value: 2.0 × 10^−6^, *n* = 6).

### Statistics

Student’s t-tests were performed using GraphPad Prism 5 software (GraphPad, La Jolla, CA). R was used for linear regression analysis of gene expression. All tests were two-sided and *p* < 0.05 was considered statistically significant. Variance was assumed to be similar between compared groups. Adjustments for multiple comparisons were applied for gene correlations across the genome (FDR). For the cell culture experiments, no sample size calculation was performed. For the AMC cohort patient data, SPSS package 24 (IBM Analytics, Armonk, NY) was used for Chi-square testing, Mann–Whitney U test, Spearman’s rank correlation, Cox proportional hazard regression modeling, Kaplan–Meier survival analysis, and log-rank test. Patients who died within 30 days after operation were excluded from survival analysis as these likely did not succumb to cancer (*n* *=* 5). Otherwise, all eligible samples were measured and no power calculation was performed. Cox proportional hazard regression was used for univariate and multivariate analyses to investigate the correlation of OS with ADAM12 and potential prognostic factors.

For the MPACT cohort, baseline ADAM12 values were categorized into undetectable (0) and detectable (> 0). The fold change of ADAM12 at cycle 2, day 1 (C2D1) from baseline (cycle 1, day 1; C1D1) was calculated by the results at C2D1 divided by the value at C1D1. These were then assigned to three groups (0: both values below detection limit; < 1 if levels decreased, >1 if values increased). Descriptive statistics summary of demographics and baseline characteristics were performed. Correlation of ADAM12 groups with categorical variables was tested using CMH statistics or Cochran-Armitage Trend Test statistics, or one-way ANOVA for continuous variables. All eligible samples were measured and no power calculation was performed. Overall survival was analyzed using Kaplan–Meier method and log-rank test. SAS 9.2 (SAS Institute Inc., Cary, NC) was used for all statistical analyses in the MPACT study.

## Electronic supplementary material

Supplementary Information

Supplemental Figure 1

Supplemental Figure 2

Supplemental Figure 3
