# Supplementary material for: ADAM12 is a circulating marker for stromal activation in pancreatic cancer and predicts response to chemotherapy
Source: Oncogenesis. 2018 Nov 16;7(11):87. doi: 10.1038/s41389-018-0096-9 (PMC6237826; doi:10.1038/s41389-018-0096-9)
Supplement: Supplementary file 1 — Supplementary Information [file 41389_2018_96_MOESM1_ESM.docx]

## SUPPLEMENTARY INFORMATION FOR

# ADAM12 is a circulating marker for stromal activation in pancreatic cancer and predicts response to chemotherapy

V.L. Veenstra, H. Damhofer, C. Waasdorp, L. B. van Rijssen, M. J. van de Vijver, F. Dijk, H. W. Wilmink, M. G. Besselink, O.R. Busch, D.K. Chang, P.J. Bailey, A.V. Biankin, H.M. Kocher, J.P. Medema, J.S. Li, R. Jiang, D.W. Pierce, H.W.M. van Laarhoven & M.F. Bijlsma

**SUPPLEMENTARY FIGURE LEGENDS**

###

### Supplementary Figure S1. ADAM12 expression correlates with survival in pancreatic cancer.

(a) Boxes indicate median with first and third quartiles of log2 transformed gene expression values from two U133 Plus 2.0 microarray datasets of pancreatic cancer patients comparing normal and tumor tissue, using the Zhang *et al*. Human Gene Chip 1.0 platform expression data (GSE28735; (1)). *n*=45 paired biopsies, ****p*<0.001. (b) Kaplan-Meier plot of patients from the GSE15471 set, dichotomized for median ADAM12 expression.

### Supplementary Figure S2. ADAM12 expression correlates with stromal activation in pancreatic cancer.

(a) Scatter plot of ADAM12 gene expression and indicated stromal activation markers in bulk patient tumors (GSE16515 (2), GSE15471 (3), GSE28735 (1)). Line indicates linear regression fit, shaded area indicates standard error confidence bounds, determined using the R linear model function. Size of dots indicates expression of epithelial marker gene KRT19. No strong inverse correlations with tumor cell content as inferred from cytokeratin 19 (KRT19) and epithelial cell adhesion molecule (EPCAM) were found. R-squared (R^2^) linear regression coefficients and statistical significance of regression are shown in tabular overview. (b) Gene set enrichment analysis (GSEA) on the GSE16515 dataset using gene ontology set ‘extracellular matrix’ as a gene signature, pancreatic stromal signature by Binkley et al. (4) Samples were dichotomized by median ADAM12 expression. NES, normalized enrichment score, FWER p, family-wise error rate multiple testing-corrected significance.

**Supplementary Figure S3.** **Plasma ADAM12 levels correlate with favorable outcome in metastatic PDAC patients.** (a) Baseline recalcified plasma samples from the entire MPACT cohort were analyzed for ADAM12 and patients were dichotomized for ADAM12 levels above or below detection (2 x SD of the OD of blanks). Kaplan-Meier survival analysis was performed, with log-rank testing. (b) Baseline (cycle 1, day 1) and follow-up (cycle 2, day 1) samples were measured and fold change was calculated. Samples with undetectable ADAM12 levels at both baseline and follow-up did not show a fold change and are indicated by the red line.

**SUPPLEMENTARY TABLES**

### Supplementary Table S1. Baseline characteristics of AMC PDAC patients (2012-2015). Asterisks indicate parameters with incomplete coverage. The stage IV patient in the resected cohort had distant positive lymph nodes and was staged as M1. IQR; interquartile range, LNR; lymph node ratio.

|  | **all** | | **resected** | | **unresectable** | |
| --- | --- | --- | --- | --- | --- | --- |
|  | *N* | median (IQR) | *N* | median (IQR) | *N* | median (IQR) |
| age (years) | 149 | 67 (61-73) | 60 | 67 (62-73) | 89 | 68 (60-73) |
| gender | 149 | male (53%) | 60 | male (58%) | 89 | male (49%) |
| stage | 149 | I (4.7%) | 60 | I (11.7%) | 89 | I (0%) |
| ­ |  | II (34.9%) |  | II (70%) |  | II (11.2%) |
|  |  | III (28.2%) |  | III (16.7%) |  | III (36%) |
|  |  | IV (32.2%) |  | IV (1.7%) |  | IV (52.8%) |
| tumor size (mm)* | 141 | 35 (25-42) | 58 | 29 (22-35) | 83 | 38 (30-47) |
| CA19 (kU/L)* | 96 | 296 (35-1907) | 27 | 43 (12-215) | 69 | 668 (77-2431) |
| LNR* | 62 | 0.14 (0-0.33) | 59 | 0.14 (0-0.32) | 3 | 1 (0.19-1) |
| ADAM12 (pg/mL) | 149 | 372 (206-830) | 60 | 268 (172-688) | 89 | 407 (246-855) |

### Supplementary Table S2. Correlations of ADAM12 serum levels and baseline clinicopathological parameters of the AMC cohort. Patients were divided into high and low ADAM12 serum level by ROC (316 pg/mL, determined on the resected cohort). CA19-9 and LNR were tested as continuous variables.

|  |  | **resected** | |  |  | **unresected** | | |  |
| --- | --- | --- | --- | --- | --- | --- | --- | --- | --- |
|  |  | low | high | Total | *P* value | Low | High | Total | *P* value |
| age |  | 34 | 26 | 60 (100%) | 0.303 | 32 | 57 | 89 (100%) | 0.416 |
| gender | male | 18 | 17 | 35 (58%) | 0.333 | 13 | 31 | 44 (49%) | 0.213 |
|  | female | 16 | 9 | 25 (42%) |  | 19 | 26 | 45 (51%) |  |
| stage | I | 3 | 4 | 7 (12%) | 0.475 |  |  |  |  |
|  | II | 26 | 16 | 42 (70%) |  | 4 | 6 | 10 (11%) | 0.948 |
|  | III | 5 | 5 | 10 (17%) |  | 11 | 21 | 32 (36%) |  |
|  | IV | 0 | 1 | 1 (1%) |  | 17 | 30 | 47 (53%) |  |
| tumor size | ≤20mm | 8 | 4 | 12 (20%) | 0.443 | 0 | 2 | 2 (2%) | 0.547 |
|  | >20mm | 25 | 21 | 46 (77%) |  | 28 | 53 | 81 (91%) |  |
| CA19-9 |  | 15 | 12 | 27 (45%) | 0.197 | 20 | 49 | 69 (78%) | 0.530 |
| LNR |  | 34 | 25 | 59 (98%) | 0.944 |  |  |  |  |

**Supplementary** **Table S3. Cox proportional hazard regression model on overall survival in the AMC cohorts.** Multivariate analysis for overall survival in PDAC patients (resected *n*=58; unresectable *n*=86). ADAM12 cut-off as for Supplementary Table 2. Dichotomization of resected patients by CA19-9 was at 41.5 kU/L, of unresectable patients at 354 kU/L.

|  |  | **resected** | |  | **unresected** | |  |
| --- | --- | --- | --- | --- | --- | --- | --- |
|  |  | HR | 95% CI | *P* value | HR | 95% CI | *P* value |
| stage | I | 1 |  |  |  |  |  |
|  | II | 1.333 | 0.24-7.32 | 0.741 | 1 |  |  |
|  | III | 2.274 | 0.21-25.03 | 0.502 | 1.148 | 0.43-3.10 | 0.785 |
|  | IV |  |  |  | 2.403 | 0.90-6.43 | 0.081 |
| tumor size | ≤20mm | 1 |  |  | 1 |  |  |
|  | >20mm | 3.77 | 0.72-19.66 | 0.115 | 185011.84 | 0-3.95E+181 | 0.953 |
| CA19-9 | low | 1 |  |  | 1 |  |  |
|  | high | 6.507 | 0.99-42.55 | 0.51 | 1.686 | 0.93-3.07 | 0.088 |
| LNR |  | 1.062 |  | 0.969 |  |  |  |
| ADAM12 | low | 1 |  |  | 1 |  |  |
|  | high | 0.643 | 0.19-2.21 | 0.48 | 1.112 | 0.62-2.01 | 0.725 |

**Supplementary Table S4.** Treatment group sizes by ADAM12 plasma level dichotomization of the MPACT cohort. (*A*) Baseline levels, and (*B*) fold change in ADAM12 levels at follow-up relative to baseline (C1D1 to C2D1).

| a |  | ADAM12 at C1D1 | |  |
| --- | --- | --- | --- | --- |
|  |  | undetectable | detectable |  |
| Treatment | Nab-paclitaxel +Gemcitabine | 45/95 (47%) | 139/277 (50%) | |
|  | Gemcitabine | 50/95 (53%) | 138/277 (50%) | |

| b |  | ADAM12 fold change | | |
| --- | --- | --- | --- | --- |
|  |  | 0 | <1 | >1 |
| Treatment | Nab-paclitaxel +Gemcitabine | 22/38 (58%) | 59/112 (53%) | 27/58 (47%) |
|  | Gemcitabine | 16/38 (42%) | 53/112 (47%) | 31/58 (53%) |

**Supplementary** **Table S5. Correlation of ADAM12 plasma levels to clinicopathological parameters of the MPACT cohort.** (a) Baseline characteristics of cohort divided by baseline ADAM12 levels are shown. NLR; neutrophil-to-lymphocyte ratio. KPS; Karnofsky performance status; #, *F*-test applied. (b) As panel A, for fold change in ADAM12 from C1D1 to C2D1. Asterisk indicated CMH tested *p*-value.

|  |  | ADAM12 at C1D1 | |  |  |
| --- | --- | --- | --- | --- | --- |
| a |  | undetectable (*N*=95) | detectable  (*N*=277) | total  (*N*=372) | CMH  *P*-value |
| NLR | ≤5 | 71 | 161 | 232 (62%) | 0.004 |
|  | >5 | 24 | 116 | 140 (38%) |  |
| CA19-9 | ≥59xuln | 48 | 132 | 180 (48%) | 0.7447 |
|  | >uln - <59xuln | 28 | 81 | 109 (29%) |  |
|  | normal | 13 | 40 | 53 (14%) |  |
| KPS at baseline | 90-100 | 65 | 161 | 226 (60%) | 0.0892 |
|  | 70-80 | 30 | 114 | 144 (39%) |  |
| liver metastasis | yes | 78 | 233 | 311 (84%) | 0.6484 |
|  | no | 17 | 44 | 61 (16%) |  |
| age (years) | <65 | 52 | 166 | 218 (59%) | 0.3760 |
|  | ≥65 | 43 | 111 | 154 (41%) |  |
| sum of longest diam at BL (cm) | least squares mean | 10.4 | 12.1 |  | 0.0526^#^ |

|  |  | ADAM12 fold change | | |  |  |
| --- | --- | --- | --- | --- | --- | --- |
| b |  | 0  (*N* =38) | <1  (*N*=112) | >1  (*N*=58) | total  (*N*=208) | Trend  *P*-value |
| NLR | ≤5 | 31 | 83 | 34 | 148 (71%) | 0.0106 |
|  | >5 | 7 | 29 | 24 | 60 (29%) |  |
| CA19-9 | ≥59xuln | 18 | 54 | 34 | 106 (51%) | 0.1852* |
|  | >uln - <59xuln | 12 | 36 | 17 | 65 (31%) |  |
|  | normal | 6 | 16 | 5 | 27 (13%) |  |
| KPS at baseline | 90-100 | 32 | 74 | 36 | 142 (68%) | 0.0325 |
|  | 70-80 | 6 | 38 | 22 | 66 (32%) |  |
| liver metastasis | yes | 28 | 93 | 50 | 171 (82%) | 0.1340 |
|  | no | 10 | 19 | 8 | 37 (18%) |  |
| age (years) | <65 | 21 | 69 | 40 | 130 (63%) | 0.1663 |
|  | ≥65 | 17 | 43 | 18 | 78 (37%) |  |
| sum of longest diam at BL (cm) | least squares mean | 8.5 | 10.4 | 13.9 |  | 0.0003^#^ |

**Supplementary** **Table S6. Univariate Cox Regression Model for the MPACT cohort.** Including the individual potentially prognostic factors.

|  |  | HR | 95% CI | *P* value |
| --- | --- | --- | --- | --- |
| age | <65 vs ≥65 | 0.86 | 0.69 -1.07 | 0.1654 |
| liver metastasis | no vs yes | 0.55 | 0.41 -0.75 | 0.0001 |
| CA19-9 at baseline | ≥59xULN vs normal | 1.36 | 0.98 -1.88 | 0.0667 |
|  | >ULN-<59xULN vs normal | 1.12 | 0.79 -1.58 | 0.5262 |
| NLR | ≤5 vs >5 | 0.43 | 0.35 -0.54 | <.0001 |
| ADAM12 baseline | 0 vs >0 | 0.71 | 0.55 -0.91 | 0.0062 |
| ADAM12 Fold change | <1 vs 0 | 1.52 | 1.00 -2.29 | 0.0478 |
|  | >1 vs 0 | 2.26 | 1.43 -3.56 | 0.0005 |
| treatment | A+GEM vs GEM | 0.81 | 0.66 -1.01 | 0.0582 |
| KPS | 90-100 vs 70-80 | 0.57 | 0.46 -0.71 | <.0001 |
| sum of longest diameter at baseline | every 5 cm increase | 1.18 | 1.11 -1.26 | <.0001 |

**Supplementary** **Table S7. Multivariate Cox Regression Model.** Including ADAM12 at baseline, treatment arm, and KPS as major factors.

|  |  | HR | 95% CI | P-value |
| --- | --- | --- | --- | --- |
| ADAM12 baseline | 0 vs >0 | 0.75 | 0.59 -0.96 | 0.0241 |
| treatment | A+GEM vs GEM | 0.77 | 0.62 -0.96 | 0.0194 |
| KPS | 90-100 vs 70-80 | 0.58 | 0.47 -0.73 | <.0001 |

**SUPPLEMENTARY REFERENCES**

1. Zhang G et al. Integration of metabolomics and transcriptomics revealed a fatty acid network exerting growth inhibitory effects in human pancreatic cancer. Clinical cancer research : an official journal of the American Association for Cancer Research. 2013;19(18):4983-93.

2. Pei H et al. FKBP51 affects cancer cell response to chemotherapy by negatively regulating Akt. Cancer Cell. 2009;16(3):259-66.

3. Badea L, Herlea V, Dima SO, Dumitrascu T, Popescu I. Combined gene expression analysis of whole-tissue and microdissected pancreatic ductal adenocarcinoma identifies genes specifically overexpressed in tumor epithelia. Hepatogastroenterology. 2008;55(88):2016-27.

4. Binkley CE et al. The molecular basis of pancreatic fibrosis: common stromal gene expression in chronic pancreatitis and pancreatic adenocarcinoma. Pancreas. 2004;29(4):254- 63.
