# Supplementary figures and images for: ADAM12 is a circulating marker for stromal activation in pancreatic cancer and predicts response to chemotherapy
Source: Oncogenesis. 2018 Nov 16;7(11):87. doi: 10.1038/s41389-018-0096-9 (PMC6237826; doi:10.1038/s41389-018-0096-9)

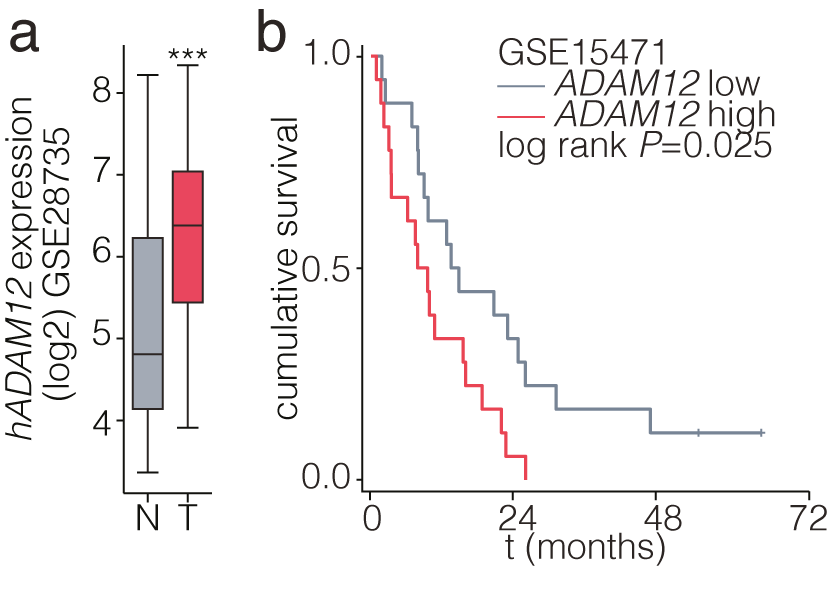

Supplement: Supplementary file 2 — Supplemental Figure 1 [file 41389_2018_96_MOESM2_ESM.tif]

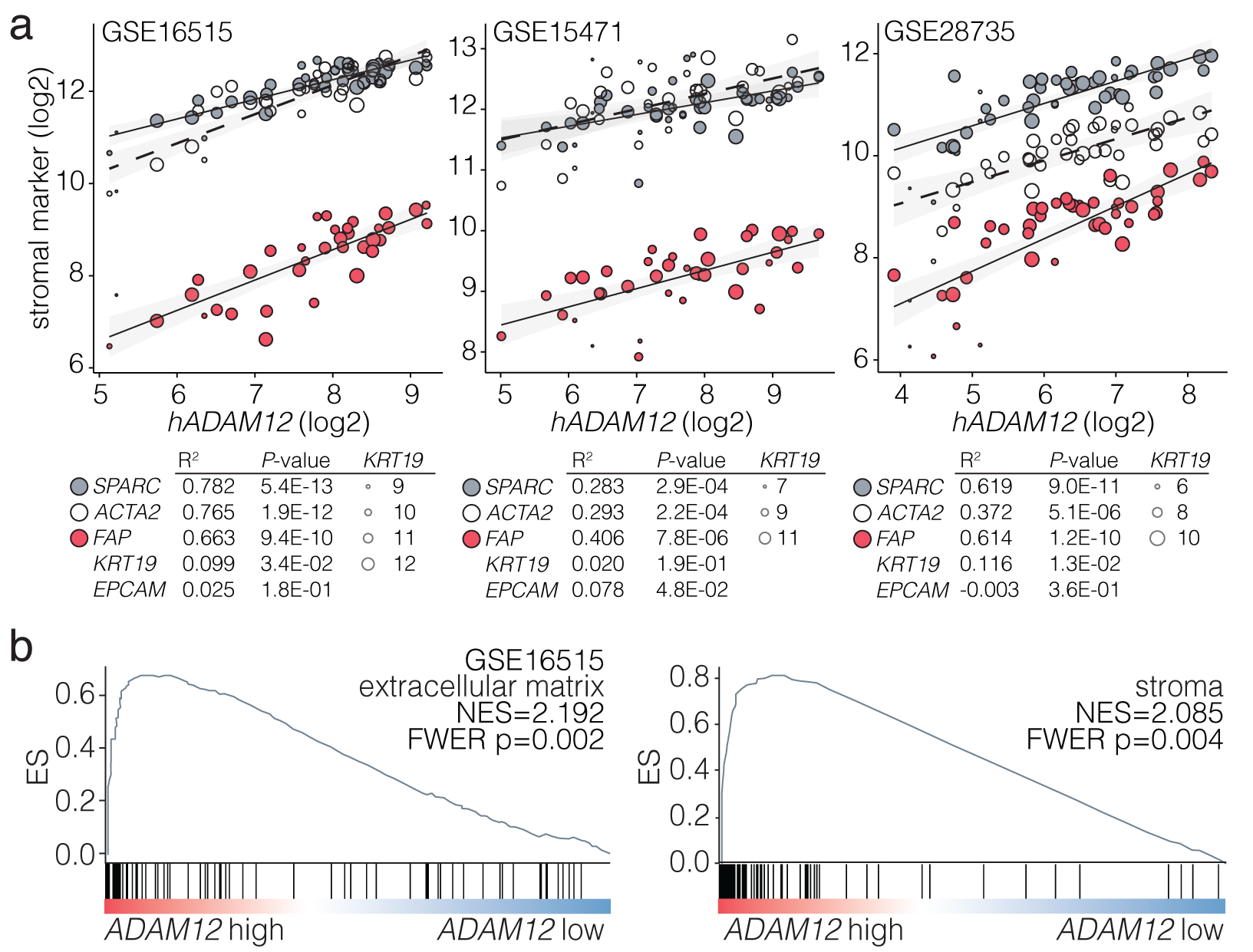

Supplement: Supplementary file 3 — Supplemental Figure 2 [file 41389_2018_96_MOESM3_ESM.tif]

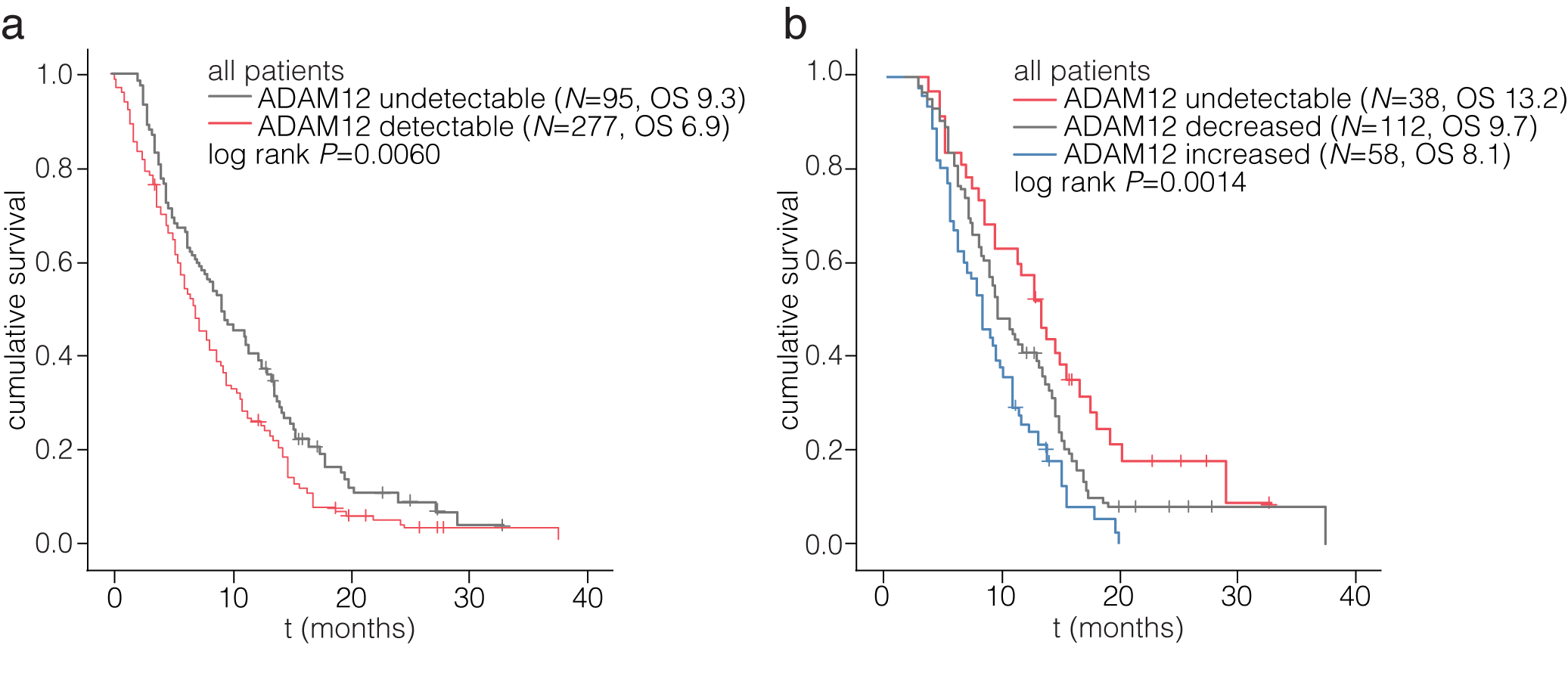

Supplement: Supplementary file 4 — Supplemental Figure 3 [file 41389_2018_96_MOESM4_ESM.tif]
